# Structure of the mannose phosphotransferase system (man-PTS) complexed with microcin E492, a pore-forming bacteriocin

**DOI:** 10.1038/s41421-021-00253-6

**Published:** 2021-04-06

**Authors:** Kai Huang, Jianwei Zeng, Xueli Liu, Tianyu Jiang, Jiawei Wang

**Affiliations:** State Key Laboratory of Membrane Biology, Beijing Advanced Innovation Center for Structural Biology, School of Life Sciences, Tsinghua University, Beijing, 100084 China

**Keywords:** Cryoelectron microscopy, Protein translocation

Dear Editor,

Bacteriocins comprise various protein classes produced and secreted by bacteria that have a toxic effect on strains closely related to the producer strain^1^. Their functions are diverse, including pore-formation, DNases, RNases, and inhibition of protein synthesis or DNA replication. Bacteriocins produced by Gram-negative bacteria are divided based on their molecular masses into colicins (high molecular mass, 30–80 kDa) and microcins (Mccs; low molecular mass, < 10 kDa)^2^. Mccs are hydrophobic peptides that are highly resistant to heat, extreme pH, and proteases with potent antibacterial activity^3^. These properties are considered essential for the role that Mcc may play in intestinal microbiota, specifically in bacterial displacements. Mccs can be classified into two classes. Class I encompasses highly posttranslationally modified very-low-molecular-mass peptides (< 5 kDa) which must cross the inner membrane and target intracellular enzymes responsible for the DNA/RNA structure or synthesis. Class II, including high-molecular-mass peptides (ranging from 5 to 10 kDa), is subdivided into two subclasses: (1) class IIa Mccs need no further posttranslational modification (MccL, MccV, and Mcc24); and (2) class IIb Mccs are linear peptides that may carry a C-terminal posttranslational modification (MccE492, MccM, MccH47, and MccI47). MccE492 (UniProt: Q9Z4N4) is a pore-forming bacteriocin produced and exported by *Klebsiella pneumoniae* RYC492^4^. MccE492 undergoes posttranslational modification with the covalent linkage of a catechol-type siderophore at the C-terminal end with the glycine–serine motif^5^ (Supplementary Fig. S1). The N-terminal leader peptide of MccE492 is cleaved^6^; the secreted, modified protein is called active MccE492, and its polypeptide backbone is referred to as MceA^7^. The C terminus of active MccE492 (siderophore) is recognized by the outer membrane catecholate siderophore receptor FepA, Fiu, and Cir of several species of Enterobacteriaceae^4^ and translocated across the outer membrane by using the “Trojan horse” strategy. Once in the periplasm, MccE492 inserts itself into the cytoplasmic membrane and stably associates with ManYZ, the inner membrane component of the mannose permease (man-PTS)^8^. Subsequently, it kills target cells through depolarization and permeabilization of their inner membrane, thereby disrupting the proton motive force^9^. MccE492-producing bacteria synthesize a 95-amino acid immunity protein (MceB), which renders the cell resistant to MccE492^10^. The predicted structure of MceB shows three hydrophobic domains and suggests an integral membrane protein, implying that MceB sequesters or blocks the entry of active MccE492. To elucidate the specific interaction between MccE492 (MceA + siderophore) and ManYZ, we sought to resolve the structure of the MceA–ManYZ complex, which is necessary for designing improved toxic molecules for attacking specific targets.

Glutathione S-transferase (GST) protein was fused to the N terminus of MceA and co-expressed with ManY and ManZ in *Escherichia coli* to use the endogenous bactericidal activity of MceA^9^. After GST affinity tag removal and size exclusion chromatography purification, peak fractions were concentrated for cryo-EM analysis (Supplementary Fig. S2). A total of 92,052 selected particles yielded a three-dimensional EM reconstruction at an overall resolution of 2.28 Å (Supplementary Figs. S3, S4 and Table S1). ManY and ManZ could be classified as CoreY (colored orange), ArmY (pale cyan), and VmotifY (cyan) and CoreZ (lime), ArmZ (pink), and VmotifZ (magenta) domains, respectively^8^ (Fig. 1a, b and Supplementary Fig. S5a, b). VmotifY and VmotifZ interlock to form the Vmotif domain. CoreY and CoreZ clamp the substrate to form the Core domain. As with the apo-form ManYZ structure^8^, the overall structure of the MccE492–ManYZ/MceA–ManYZ complex reveals a threefold symmetry axis perpendicular to the membrane, in which MccE492 and ManYZ assembled in a 3:3 ratio (Fig. 1a). In the MccE492–ManYZ structure, the mannose substrate is localized in the middle of the membrane (Fig. 1b). Therefore, the ManYZ structure in the complex is identified as the occluded conformation. When the two structures (Apo–ManYZ and MccE492–ManYZ) are superposed according to their orientation in membranes (Fig. 1a, b and Supplementary Fig. S6a, b), the transmembrane parts of Vmotifs align well (Supplementary Fig. S6c). The difference between the two structures can be described as the rearrangement of Cores relative to Vmotifs. The Core domain undergoes a rigid-body rotation of approximately 47° and a translation of approximately 11 Å when switching from the inward-open to occluded conformation, with the substrate-binding site remaining unchanged (Supplementary Fig. S6d). Apo–ManYZ has an inward-facing conformation, whereas an occluded state is captured by the bound MccE492, which confirms the previously proposed elevator mechanism for the substrate transportation of man-PTS^8^ (Supplementary Fig. S7). An extracellular loop L78Z between TM7Z and TM8Z drastically departs from the neighbor protomer to accommodate MccE492 (Supplementary Fig. S6c). Both the N and C termini of MccE492 are localized in the periplasm (Fig. 1a and Supplementary Fig. S8). The C-terminal glycine-serine motif of MccE492 (S78–S84) could not be determined in the final model due to its flexibility (Fig. 1a). Before the glycine–serine motif, a zigzag-shaped structure (L61–T77) is embedded into a pocket formed between the Core and Vmotif domains (Fig. 1c and Supplementary Fig. S9). Binding the zigzag structure of MccE492 prevents the movement of the Core domain. Therefore, this binding interferes with mannose metabolism as the cell growth assay suggested^7^. The rest of MccE492 warps around the CoreZ subdomain (Fig. 1b). Bacteriophage λ infection of *E. coli* depends on the mannose transporter in the inner membrane for DNA penetration^11^. On the basis of our structural observation and biochemical assay, the λ DNA tunnel for bacteriophage infection is localized around the oligomerization interface between Vmotif domains. Because MccE492 binds on a different site on ManYZ, away from the oligomer interface, it does not affect susceptibility to bacteriophage λ infection^7^.

**Fig. 1:**
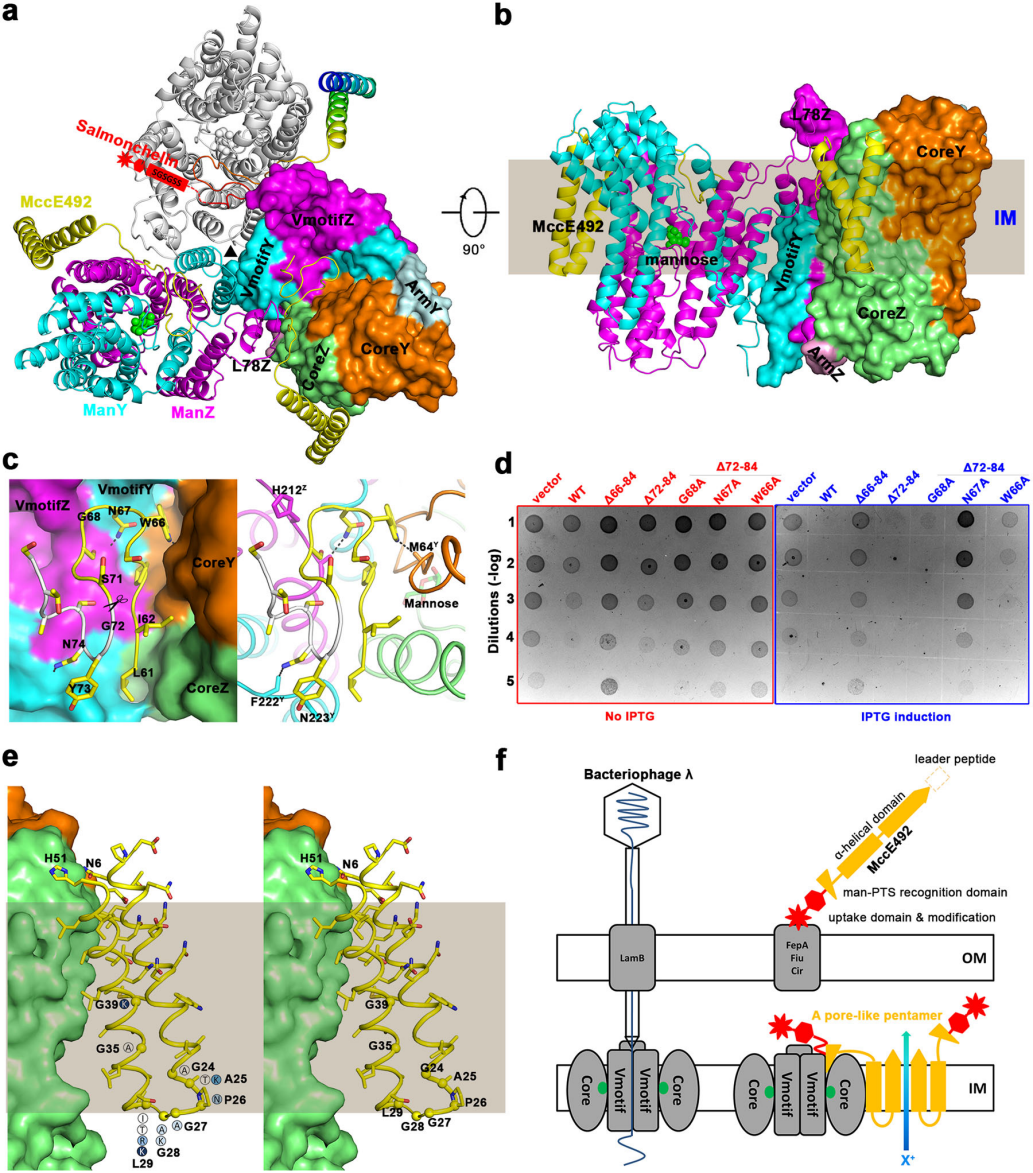
Structure of the MccE492 and ManYZ complex. **a** The structure of the MccE492–ManYZ trimer is shown in both cartoon and surface representations as viewed from the extracellular side of the membrane. MccE492s are colored yellow, except one rainbow-colored, with its N terminus in blue and its C terminus in red, and the posttranslational modification with a salmonchelin at the C-terminus is schematically shown in red. **b** The perpendicular view is shown, with the white promotor omitted for clarity. The gray rectangle shows the approximate location of the inner membrane (IM). **c** Interactions between ManYZ and man-PTS recognition domain of MccE492. Left: surface representation of ManYZ. The man-PTS recognition domain of MccE492 is shown in sticks. Δ72–84 truncation is indicated with a scissor. Right: the same orientation and representation, except the ManYZ in cartoon representation, with key residues emphasized. The hydrogen bonds are indicated with the black dash line. **d** Bactericidal activity of endogenous full-length and C-terminally truncated or mutated MceA. Serial dilutions of overnight cultures were spotted (5 µl) on LB plates without IPTG (left) or with 0.2% IPTG (right). **e** Interactions between ManYZ and α-helical domain of MccE492. The α-helical domain of MccE492 is shown in the cartoon, with side chains represented in sticks. Mutated residues in the inhibitory assay of Mcc24 are shown as spheres. Substitutions are indicated in the circles and colored white and blue in the size of the zone of inhibition. **f** Model of the mechanism of MccE492 antibacterial action. Left: it is proposed that the oligomerization interface of the ManYZ complex may be the λ DNA tunnel for bacteriophage infection. Therefore, MccE492’s association with ManYZ would not affect bacteriophage λ infection. Right: postmodification by salmonchelin-like molecules at the C terminus of MccE492 is recognized by the outer membrane proteins, FepA, Fiu, and Cir for the transportation from the extracellular medium to the periplasm. The man-PTS recognition domain is then specifically docked into the pocket between Core and Vmotif domain while ManYZ occurs in the occluded state. The man-PTS recognition domain then drives the membrane insertion of the α-helical domain. Pore formation is followed by oligomerization of additional MccE492’s.

*K. pneumoniae* RYC492 secretes siderophore catecholate modified MccE492 to exert its antibacterial action on related strains, such as *E. coli*^6^. Furthermore, *K. pneumoniae* RYC492 synthesizes a self-immunity protein MceB to protect itself from its own antibacterial product in a highly specific way (Supplementary Fig. S9a). By contrast, *E. coli* could produce Mcc24 against *Salmonella enterica serotype Typhimurium in swine*. Immunity to Mcc24 is conferred by MtfI, an integral membrane protein (Supplementary Fig. S5c). Although Mcc24 is not posttranslational modified, unlike MccE492, it is considered to have a common ancestor with MccE492, sharing 52% identity and 59% similarity. MccE492 has a modular structure, with a toxic domain at the N terminus and an uptake domain at the C terminus of the mature protein^12^ (Supplementary Fig. S9b). The C-terminal uptake module is necessary for receptor recognition and translocation across the outer membrane, whereas the N-terminal module determines antibacterial activity. The structural analysis shows that the N-terminal toxic domain can be further classified as a man-PTS recognition domain (mPRD) and an α-helical domain. The mPRD has two conserved discrete regions (Supplementary Fig. S9b). Gly52–Pro57 in the region I float away from the surface of ManZ, owing to the anchoring effect of His51 and Ile58 (Supplementary Fig. S9c). Therefore, the corresponding interacting residues on ManZ are not conserved. Starting from Ile58, region I of mPRD clings onto ManZ tightly because of its hydrophobic effect. Region II (G63–S71) forms a hairpin structure and specifically interacts with ManYZ, which is proposed to be the principal component to anchor MccE492 to the receptor.

The endogenous expression of only MceA exhibits bactericidal activity, targeting the inner membrane^9^. This endogenous MceA bactericidal assay has been used to test the effects of wild-type MceA or various mutants of MceA. In the absence of IPTG, all vectors almost do not affect colony formation size (left on Fig. 1d). On IPTG induction, WT and Δ72–84 confer strong toxicity (right on Fig. 1d). Although several hydrogen bonds, such as Asn74 to the carbonyl of Phe222^Y^ and Tyr73 to OD1 of Asn223^Y^ (hereafter, superscripts Y/Z after residue number designate ManY/ManZ), contribute to additional interactions between MceA and ManYZ (Fig. 1c), the whole uptake domain (72–84) is absent in Mcc24 (Supplementary Fig. S9b), which confirms the structural observation that this region does not determine the specificity of MceA. Starting from Ser71 N terminal to Ser65 is a hairpin structure, embedded between Core and Vmotif domains. Trp66 forms a hydrogen bond with the carbonyl group of Met64^Y^ (Fig. 1c). Similarly, Asn67 of MccE492 is hydrogen-bonded to the carbonyl group of Trp210^Z^. The mutant N67A of Δ72–84 abolishes antibacterial activity completely, whereas W66A or G68A of Δ72–84 exerts minor bactericidal effects. Further truncation of MceA until W66 (Δ66–84) leads to the loss of its antibacterial activity, which is confirmed by the fact that Mcc24 truncation at the corresponding Trp70 (Trp66 in MccE492) eliminated Mcc24 inhibition completely^13^. Therefore, the hairpin structure of Ser65–Ser71 specifically determines MceA antibacterial activity. The rest of mPRD is just a linker peptide for the α-helical domain, wrapping around the hydrophobic surface of ManZ.

The α-helical domain has a helix-turn-helix motif, and the V-shaped two helical bundles display amphipathic characters, one hydrophobic and other hydrophilic (Supplementary Fig. S10). The hydrophobic face of the α-helical domain is in contact with the ManZ hydrophobic face, except for the two residues His51 and Asn6 (Supplementary Fig. S10a), which anchor the helical ends on the receptor through electrostatic interactions. The opposing face of the α-helical domain is mainly negatively charged, and it is proposed for cationic ion transmission. Mcc24 and MccE492 are homologous bacteriocins with a sequence identity of 52%, and all mutation data on Mcc24 by Frana^13^ could be explained with the MccE492 structure. Three mutants with lysine substitutions (A25K, L29K, and G39K) completely abolished Mcc24 inhibitory activity and were among the lowest in the levels of membrane permeabilization. Ala25 and Leu29 are nonpolar amino acids and localized around the turn region of the helix-turn-helix motif. These mutations may affect the transmembrane capability of the α-helical domain. The distance between the two helices in the helix-turn-helix motif reaches the minimum at Gly39; therefore, any substitution other than glycine would affect or disrupt the helix-turn-helix motif. Furthermore, a conserved GAAGG^39^A motif was identified in MccH47, MccL, Mcc24, and ColV indicating that the toxic domain of all these Mccs forms a V-shaped helical bundle. Because of the amphipathic properties of the α-helical domain, five MccE492s might form the pentameric, pore-like structure through oligomerization^14^ similar to most pore-forming toxins and bacteriocins. In the pore formation process, the first MccE492 is specifically recognized by ManYZ. Later, sequential oligomerization might occur, which can either form a partially formed but active pore or form complete pores. Additional studies studying the mechanism of pore formation as well as the immunity function of MceB are required.

MccE492 as a precursor contains four domains: a leader peptide, an α-helical domain, mPRD, and an uptake domain (Fig. 1f). After that, a linear trimer of N-(2,3-dihydroxybenzoyl)-l-serine is anchored at the C terminus (serine 84) by β-d-glucose. After the cleavage of the leader peptide by the export complex, the active extracellular MccE492 is recognized by the outer membrane catecholate siderophore receptors FepA, Fiu, and Cir (Fig. 1f). The mechanism through which the C terminus of MccE492 is recognized by catecholate siderophore receptors is called the “Trojan horse” strategy^15^ because the C-terminal structure mimics bacterial elements, which are recognized by the respective receptors and translocated across the outer membrane. The C-terminal uptake module can be exchanged^12^. Once in the periplasm, the mPRD blocks the man-PTS complex. Then, the α-helical domain penetrates the inner membrane to form a V-shaped helix-turn-helix motif. Using ManYZ, an E492 protomer is inserted into the membrane from a soluble state to a transmembrane conformation, which is a fundamental step in pore formation. Once bound to the membrane, pore formation relies on the subsequent insertion of the extra MccE492 into the membrane. This pore channel dissipates the proton motive force of susceptible cells to exert antibacterial action. MccE492-producing bacteria synthesize an immunity protein, MceB, which renders the cell resistant to Mcc. MceB, a putative three-transmembrane helical membrane protein (Supplementary Fig. S5c), may isolate the oligomerization surface of the ManYZ-bound MccE492 promoter and prevent further oligomerization and pore formation (Supplementary Fig. S11), because MceB does not block MceA membrane insertion or prevent MceA from interacting with ManYZ^7^. It is proposed that the λ DNA tunnel for bacteriophage infection is localized around the oligomerization interface between Vmotif domains (Fig. 1f). Therefore, susceptibility to bacteriophage lambda infection is unaffected^7^. Finally, the complex structure is vital to understand the mechanism of action of Mccs and to promote new technological developments.

## Supplementary information

Supplementary Information

## Data Availability

The cryo-EM map and the structure have been deposited to the Electron Microscopy Data Bank (EMD-30923) and the Protein Data Bank (PDB: 7DYR), respectively.
