# Supplementary material for: Structure of the mannose phosphotransferase system (man-PTS) complexed with microcin E492, a pore-forming bacteriocin
Source: Cell Discov. 2021 Apr 6;7:20. doi: 10.1038/s41421-021-00253-6 (PMC8021565; doi:10.1038/s41421-021-00253-6)
Supplement: Supplementary file 1 — Supplementary Information [file 41421_2021_253_MOESM1_ESM.docx]

**Supplementary Information for**

Structure of the mannose phosphotransferase system (man-PTS) complexed with microcin E492, a pore-forming bacteriocin

Kai Huang^#^, Jianwei Zeng^#^, Xueli Liu^#^, Tianyu Jiang^*^, Jiawei Wang^*^

This file includes:

Materials and methods

Supplementary Figs. S1 to S11

Supplementary Table S1

# Materials and methods

**Protein expression and purification**

For protein expression, a gene encoding polypeptide core of MccE492 (MceA) was cloned into pQlinkG, with a Glutathione S-transferase (GST) protein in the N-terminus and a TEV protease cleavage site after the N-terminal GST. Similarly, Genes encoding ManY and ManZ were cloned into pQlinkN without any tag. Then all of them were combined by ligation-independent cloning.

Co-expression of MccE492-ManYZ was induced in *E. coli* knockout BL21 (DE3) by 0.5 mM isopropyl b-D-thiogalactoside (IPTG) when the cell density reached a D600 of 1.2. After growth overnight at 18 ℃, the cells were harvested, homogenized in buffer containing 25 mM Tris-HCl pH 8.0 and 150 mM NaCl, and disrupted by sonication. Cell debris was removed by low-speed centrifugation (12000g) for 10 min. The supernatant was applied to ultracentrifugation at 150,000 g for 1 h. The membrane pellets were collected and homogenized in the buffer (25 mM Tris pH 8.0, 150 mM NaCl and 2mM mannose), and then solubilized with 2% (w/v) n-dodecyl-b-D-maltoside (DDM, Anatrace) at 4 ℃ for 2 h. The insoluble fraction was precipitated by ultracentrifugation (150,000 g) for 30 min at 4 ℃. The supernatant was applied to Glutathione Sepharose 4B resin (GS4B, GE Healthcare). The resin was then rinsed with the buffer containing 25 mM Tris pH 8.0, 150 mM NaCl, 2 mM mannose, and 0.02% DDM three times. The protein was eluted with the elution buffer containing 50 mM Tris pH 8.0, 150 mM NaCl, 2 mM mannose, and 0.02% DDM and 7 mM glutathione. The protein complex was incubated with 2 ml TEV protease (1 mg/ml) overnight at 4 ℃ and further concentrated to 2 mL, then the protein was applied to Superdex 200 10/300 GL (SD200, GE Healthcare) pre-equilibrated with buffer containing 25 mM Tris pH 8.0, 150 mM NaCl, 2 mM mannose and 0.07% digitonin. Peak fractions were collected. For testing MccE492, the anti-MceA rabbit antiserum (GL Biochem (shanghai) Ltd.) was raised against a C-terminal fragment of MceA (SGSGYNSATSSSGSGS; synthesized at GL Biochem (shanghai) Ltd.).

**Cryo-EM data acquisition**

For negative staining, an aliquot of 4 μl sample containing 0.01mg/ml purified MccE492-ManYZ complex was applied on a carbon film grid (Beijing XXBR Technology Co. Ltd) after plasma glow discharge. The grids were stained in Uranyl acetate (2% w/v) and stored at room temperature. Negatively stained sample was imaged on a Tecnai Spirit Bio TWIN microscope (Thermo Fisher) operating at 120 kV to verify the sample quality.

Aliquots of 4 μl concentrated MccE492-ManYZ complex were loaded onto glow-discharged holey carbon grids (Quantifoil Cu R1.2/1.3, 300 mesh, C-Flat Cu R1.2/1.3, 400 mesh). Grids were blotted for 3.0 s and plunge-frozen in liquid ethane cooled by liquid nitrogen using Vitrobot Mark IV (Thermo Fisher) at 8 °C and with 100% humidity. The grids sample quality was verified with a Tecnai Arctica 200-kV electron microscope equipped with a Falcon III camera (Thermo Fisher). The verified grids with optimal ice thickness and particle density were transferred to a Titan Krios electron microscope (Thermo Fisher) operating at 300 kV and equipped with a Gatan Gif Quantum energy filter (slit width 20 eV). Micrographs were recorded using a K2 Summit counting camera (Gatan Company) in super-resolution mode with a nominal magnification of 130,000×, resulting in a calibrated pixel size of 0.53 Å. Each stack of 32 frames was exposed for 5.6 s, with an exposing time of 0.175 s per frame. The total dose for each stack was about 50 e− per Å2. AutoEMation^1^ was used for fully automated data collection. All 32 frames in each stack were aligned and summed using the whole-image motion correction program MotionCorr2^2^ and binned to a pixel size of 1.06 Å, and dose weighting was performed^3^. The defocus values were set from −1.0 to −2.0 μm and were estimated by Gctf^4^.

**Cryo-EM image processing**

The EM data processing procedure for the MccE492-ManYZ complex is outlined in Supplementary Fig. S3. In brief, all particles were automatically picked using Gautomatch (developed by Kai Zhang, https://www2.mrc-lmb.cam.ac.uk/research/locally-developed-software/zhang-software) and REION-3.0^5^ from 3638 Micrographs. All subsequent 2D and 3D classifications and refinements were performed using RELION-3.0. Multiple rounds of reference-free 2D classification were performed to remove ice spots, contaminants and aggregates, yielding a total of 1,419,964 good particles. The particles were local defocus corrected with Gctf^4^ and then processed with a global search K = 1 procedure with previous ManY_3_Z_3_ (EMDB: EMD-9906) low pass filtered to 60 Å as the initial model. The dataset from the last iteration were subject to local search 3D classification with 4-10 classes, and with an angular sampling step of 3.7° and searching range of 15°. A total of 764,524 good particles were combined and re-extracted with box size of 256, which yielded respective reconstructions at 2.83 Å with C3 symmetry and 3.12 Å without symmetry.

To reduce the dataset for final reconstruction, local search 3D classifications were executed again with 4-9 classes. Among these classes, one classes dataset contain 275,174 particles yielded reconstructions at 2.74 Å with C3 symmetry. A total of 426,108 good particles from local search were combined and subject to another local search 3D classification with 4 classes and with a mask of MccE492-ManYZ complex applied. A dataset containing 228,749 particles give rise to reconstructions at 2.77 Å with C3 symmetry and 2.95 Å without symmetry. Four additional rounds of local search 3D classification resulted in 92,076 particles improved the final resolution at 2.64 Å with C3 symmetry and 2.83 Å without symmetry. Further CTF refine together with Bayesian polishing in RELION-3.0-beta or RELION-3.1^6^ improved the final resolution at 2.34 Å with C3 symmetry and 2.46 Å without symmetry. Additionally, the resolution was improved to 2.28 Å with C3 symmetry and 2.41 Å without symmetry after optimized with larger box size of 400 pixels.

Reported resolutions were calculated on the basis of the FSC 0.143 criterion^7^, with a high-resolution noise substitution method^8^. Prior to visualization, all density maps were corrected for the modulation transfer function (MTF) of the detector and sharpened by applying a negative B-factor that was estimated using automated procedures^7^. Local resolution variations were estimated using ResMap^9^.

**Model building and structure refinement**

The 2.28 Å reconstruction map was used for *de novo* model building in EMBuilder^10^, and adjusted manually in COOT^11^. Structure refinements were carried out by PHENIX in real space with secondary structure and geometry restraints to prevent structure overfitting^12^. Statistics of 3D reconstruction and model refinement can be found in Table S1. All structure figures were prepared using PyMol^13^.

**Bactericidal activity of endogenous full-length and C-terminally truncated or mutated MceA**

Cultures of *E. coli* XL-1 Blue cells harboring plasmid pRSF (vector), WT (MceA without signal sequence), Δ66-84, Δ72-84, or mutants on Δ72-84 (G68A, N67A, W66A) were grown overnight. Both wild-type MceA and mutants contain no tag. Bactericidal activity of endogenous full-length and C-terminally truncated or mutated MceA are measured by serial dilutions. Wild-type MceA or any of the mutants were grown at 37 ℃ in LB broth supplemented with kanamycin (30μg/mL) overnight, the concentrations of the bacteria were measured by spectrophotometer OD600 and adjusted to A600 of ~1.0. After calculation, the concentration of the bacteria used for the assay were adjusted basically to the same. Serial dilutions 1:10 in LB medium containing the appropriate antibiotics kanamycin of overnight cultures (from 10-1 to 10-5) of XL-1 Blue with pRSF (vector), WT, Δ66-84， Δ72-84, G68A, N67A or W66A were spotted (5 μL) on LB plates supplemented with the appropriate antibiotics kanamycin without IPTG or with 0.2% IPTG and incubated overnight at 37 ℃。

**Supplementary Figures**

**
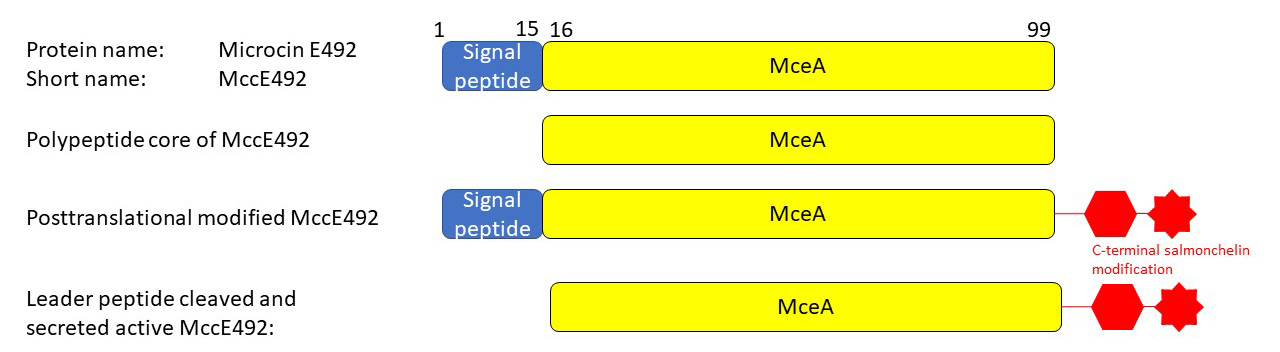
**

1. **Modular structure of microcin E492 (MccE492)**

**
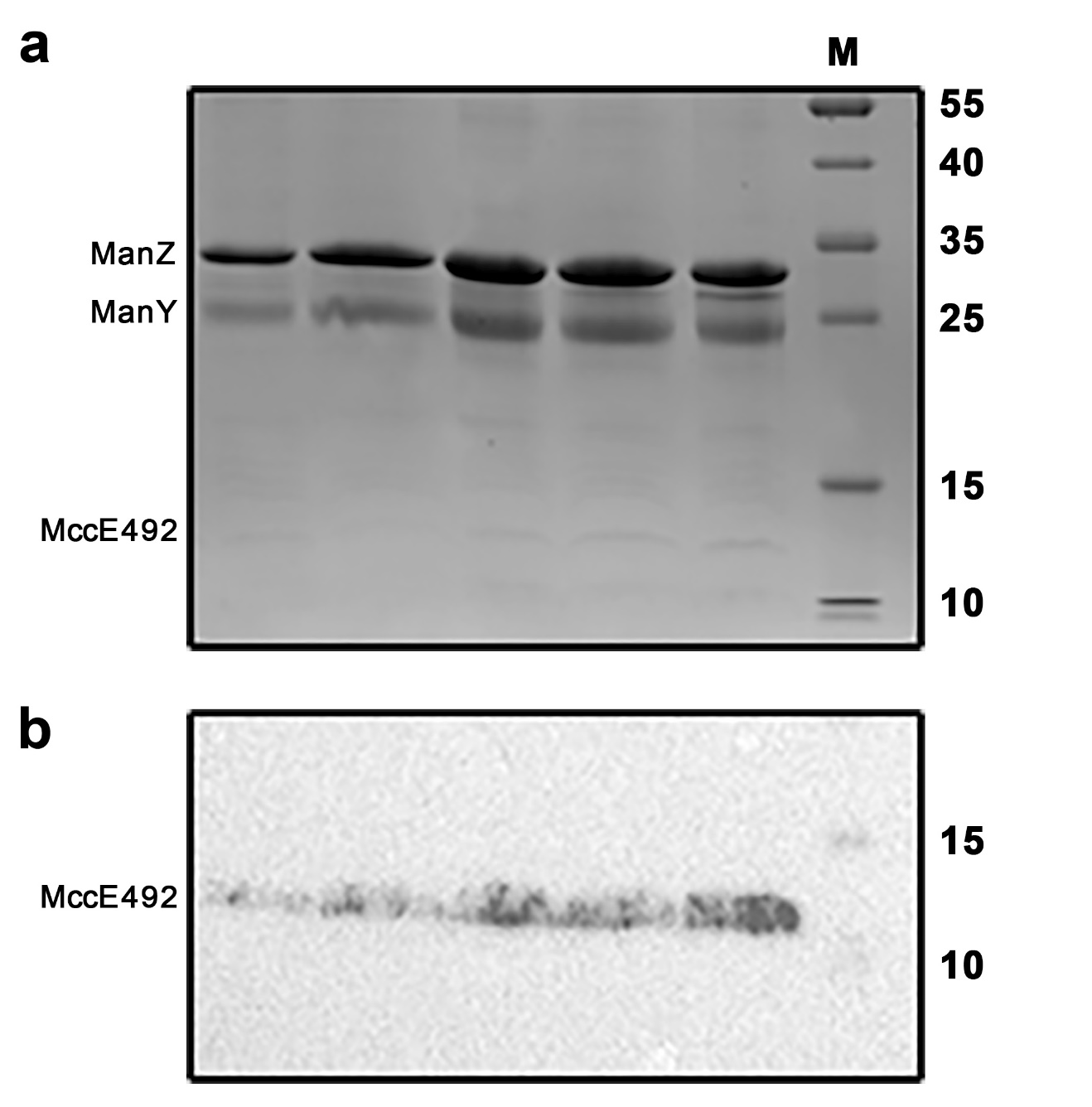
**

1. **Expression and purification of the MccE492-ManYZ complex.**

**a** The SDS-PAGE of tandem affinity purification of the MccE492-ManYZ complex visualized by coomassie blue staining. **b** Identification of MccE492 in the eluent by western blotting. Anti-MceA antibodies were applied to detect MccE492.

1. **Flowchart for cryo-EM data processing of the MccE492-ManYZ complex.**

**
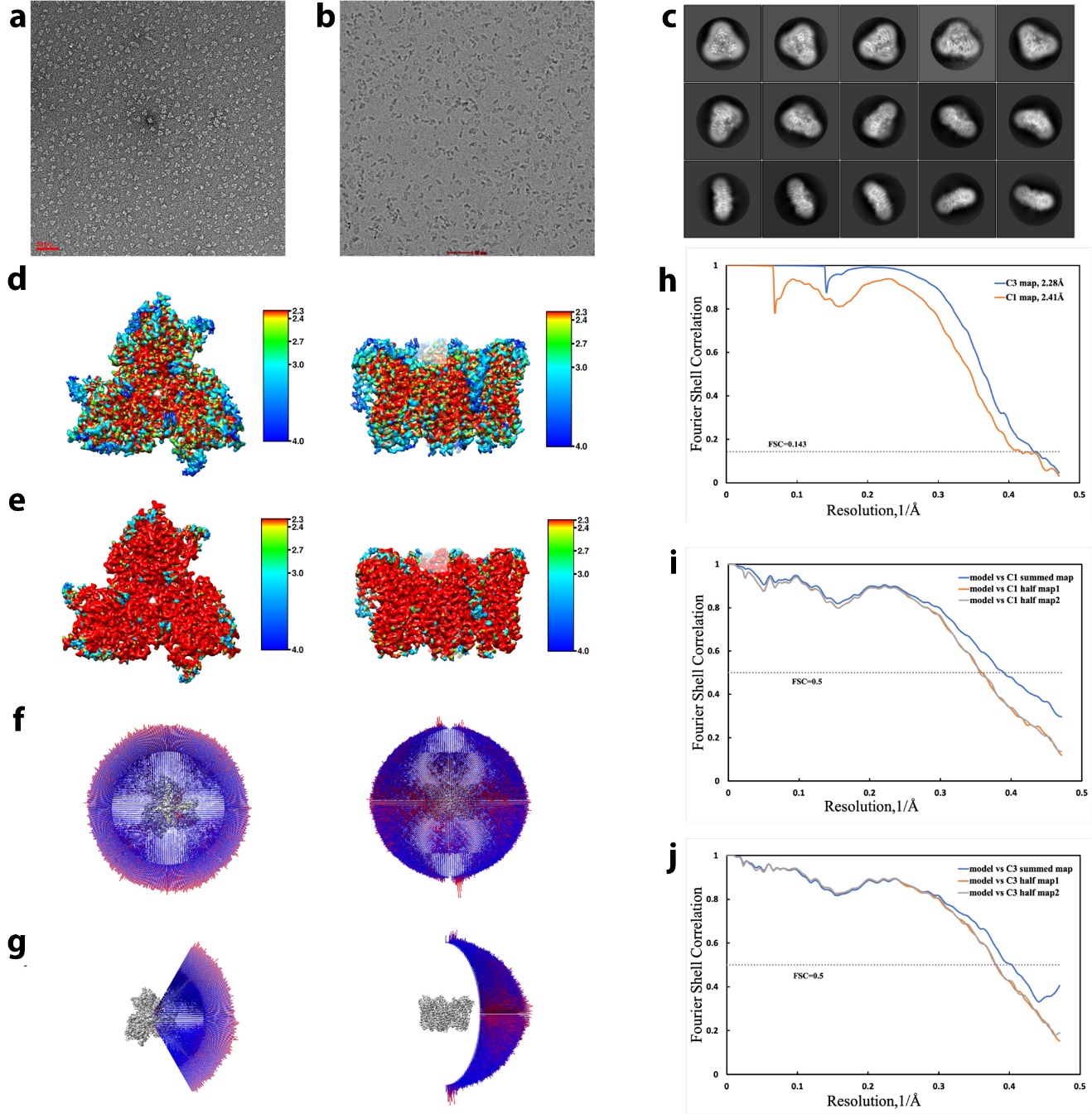
**

1. **Cryo-EM analysis of the MccE492-ManYZ complex.**

**a** A representative negative-staining EM micrograph, scale bar = 50 nm. **b** A representative cryo-EM micrograph, scale bar = 50 nm. **c** Representative 2D class averages. **d** Local resolution map for the 3D EM reconstruction C1 map. **e** Local resolution map for the 3D EM reconstruction C3 map. **f** Angular distribution of the particles of the final reconstruction C1 map. **g** Angular distribution of the particles of the final reconstruction C3 map. **h** The gold-standard Fourier shell correlation (FSC) curves for the 3D reconstructions. **i** Validation of the final structure models vs C1 map. **j** Validation of the final structure models vs C3 map.

**
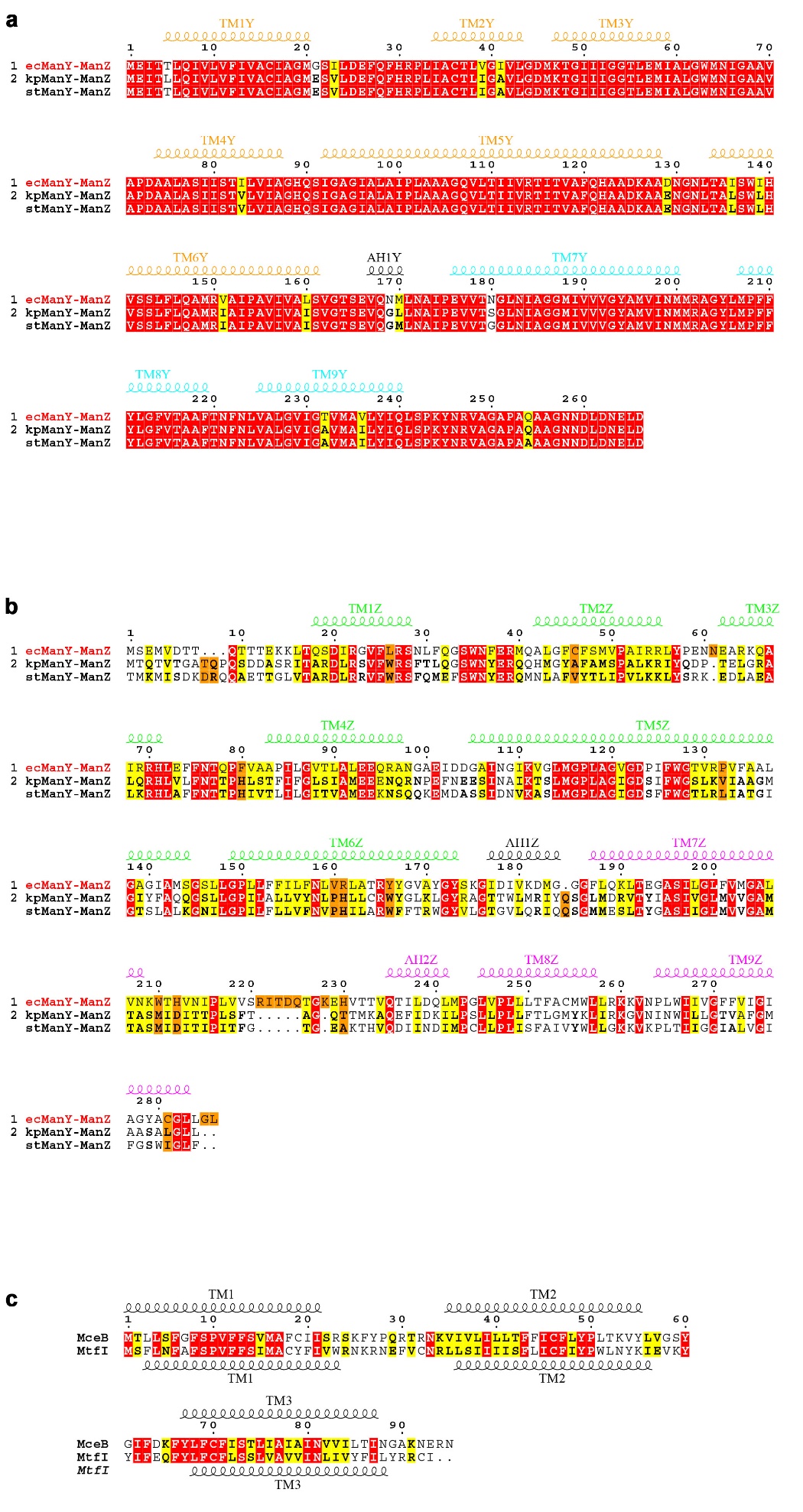
**

1. **Sequence alignment of ManY and ManZ from three different species and of immunity proteins from *Escherichia coli* and *Klebsiella pneumoniae*.**

**a,b** Secondary structural elements are indicated above the sequence alignment. The sequences were aligned with ClustalW^14^. The listed homologs include ManYZ from *Escherichia coli*, *Klebsiella pneumoniae*, and *Salmonella typhimurium.* **c** The predicted secondary structure elements of MceB and MtfI are indicated above and below the sequence alignment.

**
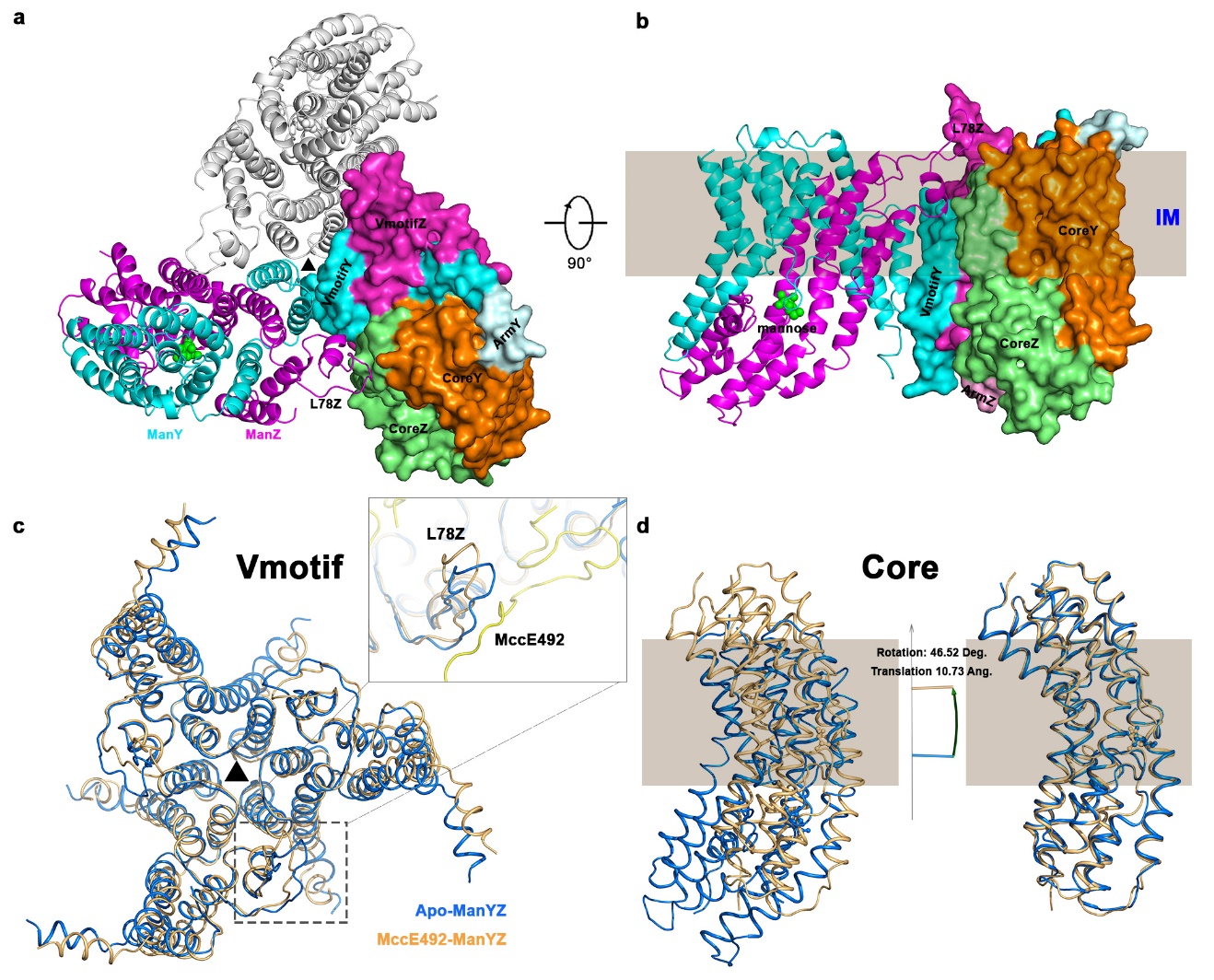
**

1. **Structural comparison of the MccE492-ManYZ complex with the apo-ManYZ (PDB code: 6K1H).**

**a, b** For comparison, apo-ManYZ trimer structure^15^ is represented in the same schemes as that in Fig. 1a (top view) and 1b (side view). **c** Structure comparison of the Vmotif domains between apo-ManYZ (marine) and MccE492-ManYZ (lightorange). **d** Structure comparison of the Core domains between apo-ManYZ and MccE492-ManYZ.


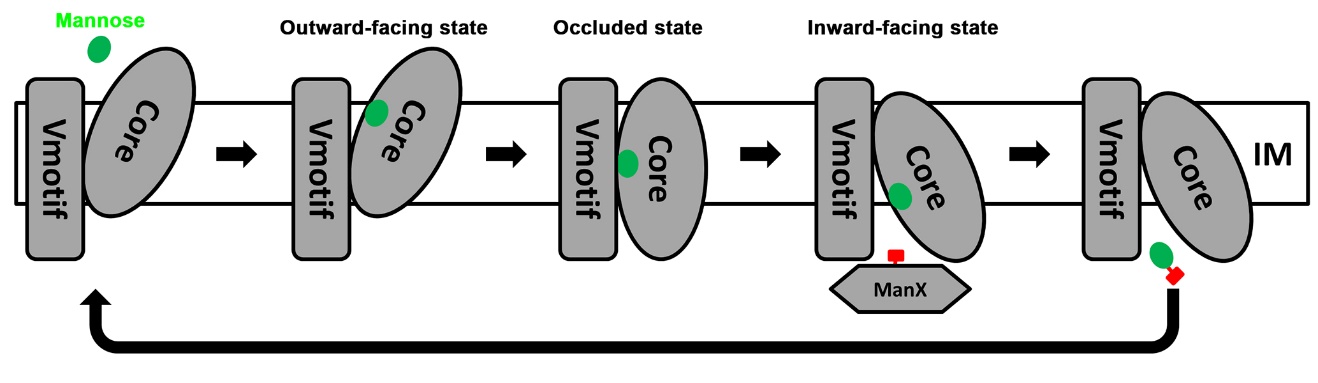


1. **Conformation changes in the substrate transport cycle of the mannose permease.** Schematic representation of the transport cycle which is divided into five conformational states with the substrate (green) and the phosphate (red).

**
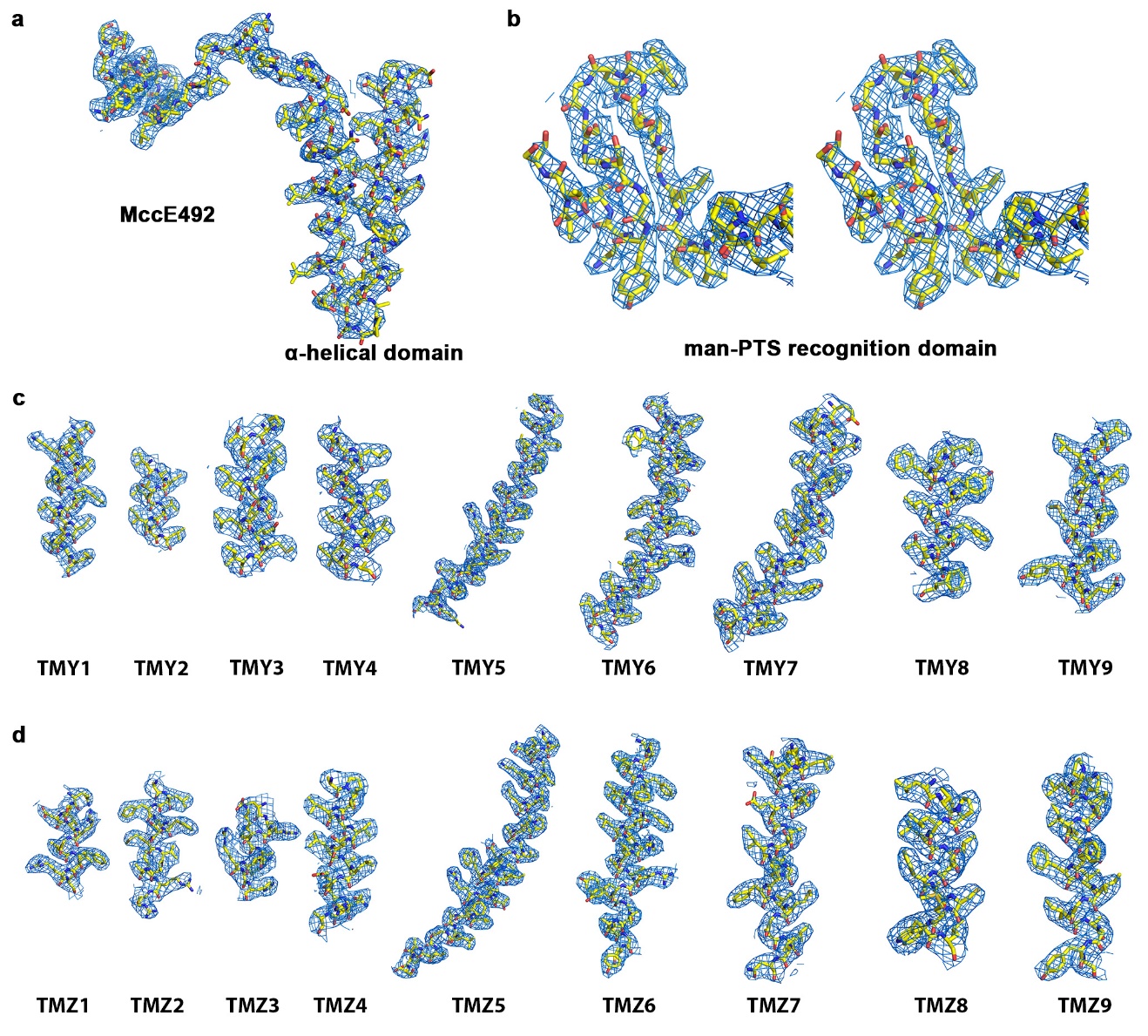
**

1. **Representative EM maps at 5σ level.**

**a** The overall EM map for MccE492. **b** Stereoview of EM map for the C terminus of MccE492. **c** EM map of different portions of the transmembrane helices of ManY. **d** EM map of different portions of the transmembrane helices of ManZ.

**
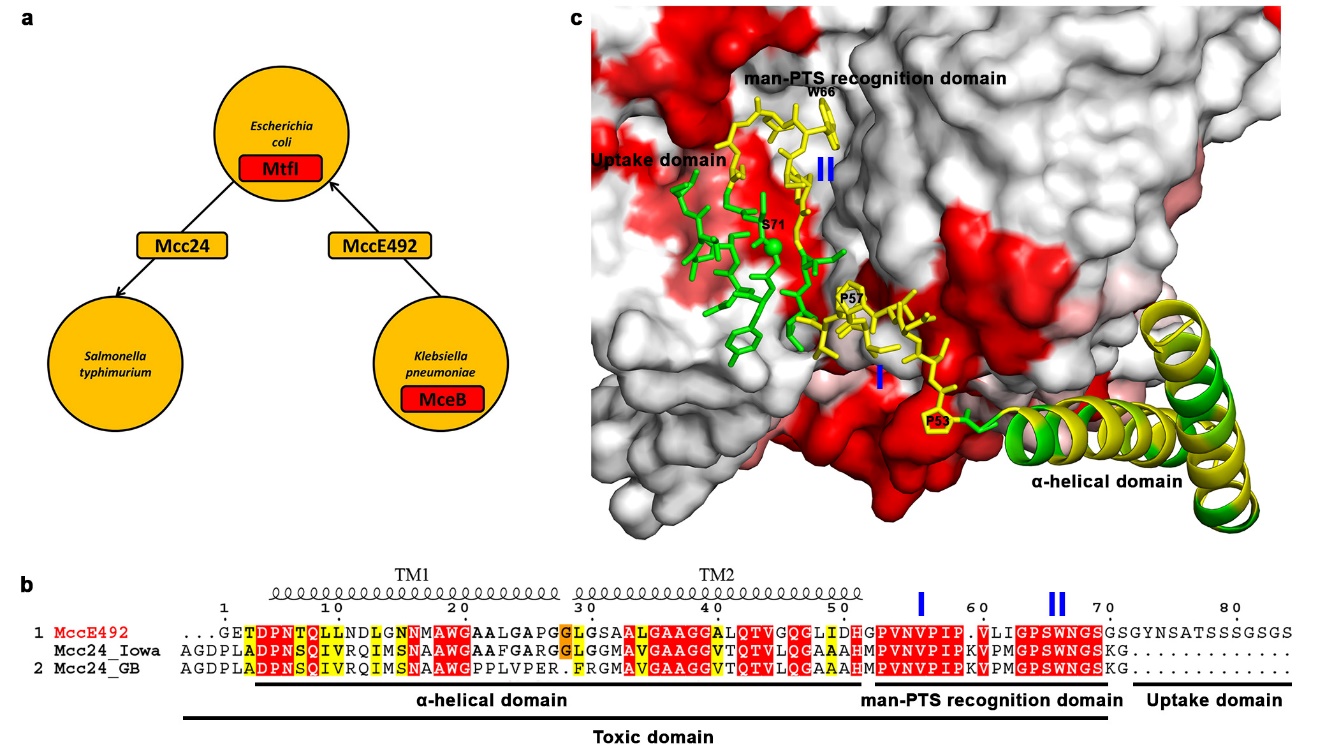
**

1. **Modular structure of MccE492 and Mcc24 peptides.**

**a** MccE492, produced by *Klebsiella pneumoniae RYC492*, causes depolarization of the *Escherichia coli* cytoplasmic membrane. Bacteria producing MccE492 synthesize an immunity protein MceB, which is also localized in the inner membrane. Mcc24 is an antimicrobial peptide produced by *Escherichia coli* with activity against *Salmonella typhimurium*. Immunity to Mcc24 is conferred by MtfI. **b** Although Mcc24 is not posttranslational modified, unlike MccE492, it is considered to have a common ancestor with MccE492, sharing 52% identity and 59% similarity. Two types of sequences exist for Mcc24: Mcc24_Iowa^16^ and Mcc24_GB (Acc # U47048), which are different in three guanine insertions at 117, 135, and 145 nucleotide positions. Differences in Mcc24_Iowa resulted in an altered predicted amino acid sequence that closely aligned with related MccE492. Mcc24_Iowa will be referred hereafter as Mcc24’s sequence. Both MccE492 and Mcc24 could exert the antibacterial function against *E. coli*, except the siderophore modification required by MccE492 across the outer membrane. Alignments of the amino acid sequences of MccE492, Mcc24_Iowa (with three guanine insertions in the GenBank sequence)^16^, Mcc24_GB (Acc # U47048) are shown with secondary structural elements indicated above the sequence alignment. Conserved amino acids are colored red and yellow in decreasing degrees of conservation. Lines indicate α-helical domain, man-PTS recognition domain, uptake domain. **c** Surface representation of ManYZ colored by conservation with a red-white gradient for variable to conserved residues. MccE492 is shown in sticks colored by conservation between MccE492 and Mcc24 with a green-yellow gradient for variable to conserved residues. The green sphere indicates the amino acid beyond which all the C-terminal residues are missed in Mcc24.


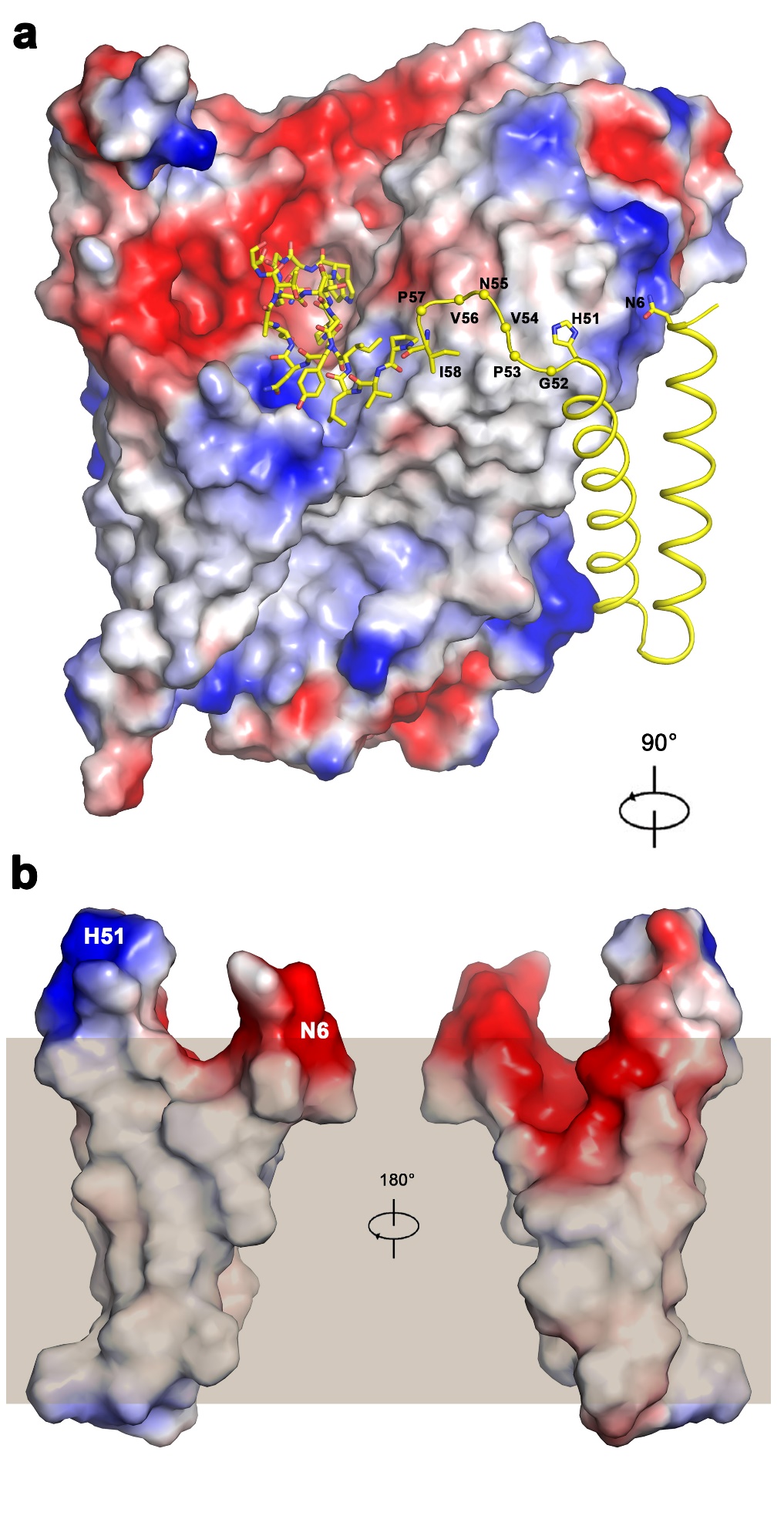


1. **Electrostatic surface potential in the MccE492-ManYZ complex.**

**a** The α-helical domain of MccE492 is shown in cartoon, except two residues (His51 and Asn6) shown with side chains. The man-PTS recognition domain and uptake domain are shown in sticks, with some residues shown as spheres for clarity. **b** Electrostatic surface potentials of α-helical domain.


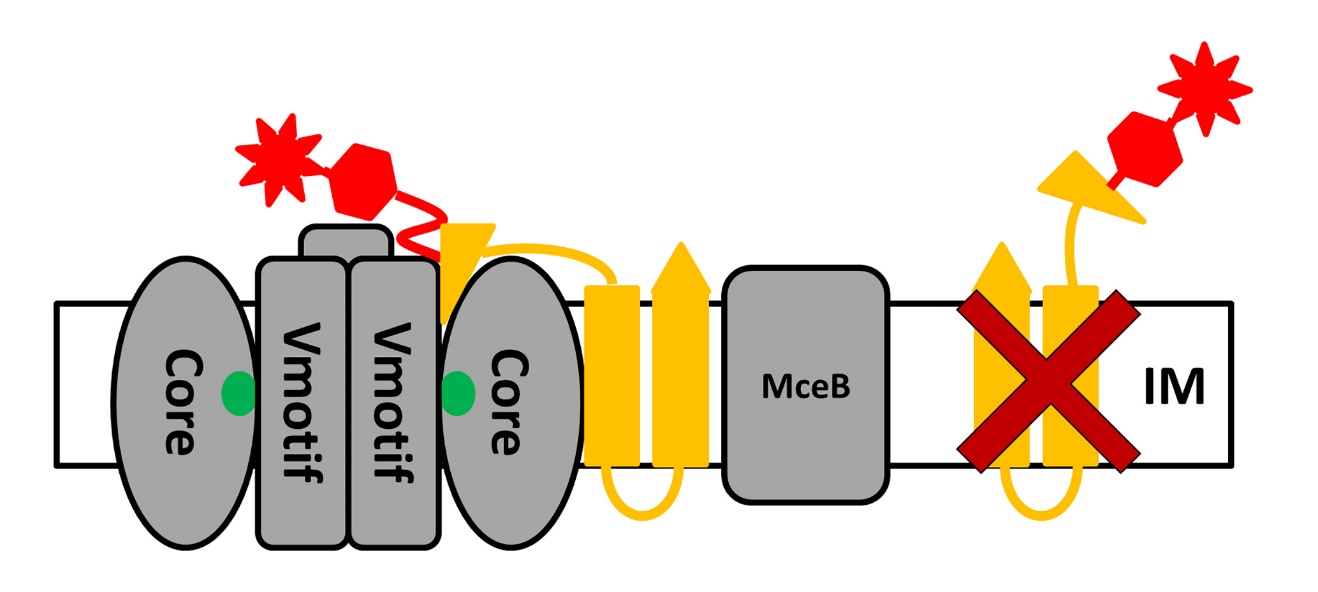


1. **Proposed mechanism of MceB immunization.**

Bacteria producing MccE492 synthesize an immunity protein MceB, which interferes with the formation of toxic pores.

**Supplementary Tables**

1. **Cryo-EM data collection, refinement and validation for the MccE492-ManYZ complex.**

|  |  |
| --- | --- |
| Data collection and processing |  |
| Voltage (kV) | 300 |
| Electron exposure (e^−^/Å^2^) | 50 |
| Defocus range (μm) | -1.0 ~ -2.0 |
| Pixel size (Å) | 1.061 |
| Symmetry imposed | C3 / C1 |
| Final dataset (# of particles) | 92,052 |
| Map resolution (Å) FSC_0.143_ | 2.28 Å (C3) / 2.41 Å (C1) |
| Refinement |  |
| Initial model used | EMD-9906 |
| Map-sharpening *B* factor (Å^2^) | -28.4 (C3) / -19.8 (C1) |
| Model validation |  |
| R.m.s. deviations |  |
| Bond lengths (Å) | 0.007 (C3) / 0.005 (C1) |
| Bond angles (°) | 0.714 (C3) / 0.674 (C1) |
| MolProbity score |  |
| Clash score | 6.49 (C3) / 4.82 (C1) |
| Poor rotamers (%) | 4.21 (C3) / 3.41 (C1) |
| Ramachandran plot (%) |  |
| Favored | 93.97 (C3) / 94.43 (C1) |
| Allowed | 5.87 (C3) / 5.35 (C1) |
| Outliers | 0.17 (C3) / 0.23 (C1) |
| Model to map fit CC | 0.89 (C3) / 0.89 (C1) |

**References and Notes**

1 Lei J, Frank J. Automated acquisition of cryo-electron micrographs for single particle reconstruction on an FEI Tecnai electron microscope. *Journal of structural biology* 2005; **150**:69-80.

2 Zheng SQ, Palovcak E, Armache JP, Verba KA, Cheng Y, Agard DA. MotionCor2: anisotropic correction of beam-induced motion for improved cryo-electron microscopy. *Nat Methods* 2017; **14**:331-332.

3 Grant T, Grigorieff N. Measuring the optimal exposure for single particle cryo-EM using a 2.6 Å reconstruction of rotavirus VP6. *Elife* 2015; **4**:e06980.

4 Zhang K. Gctf: Real-time CTF determination and correction. *Journal of structural biology* 2016; **193**:1-12.

5 Zivanov J, Nakane T, Forsberg BO *et al.* New tools for automated high-resolution cryo-EM structure determination in RELION-3. *Elife* 2018; **7**.

6 Zivanov J, Nakane T, Scheres SHW. Estimation of high-order aberrations and anisotropic magnification from cryo-EM data sets in RELION-3.1. *IUCrJ* 2020; **7**:253-267.

7 Rosenthal PB, Henderson R. Optimal determination of particle orientation, absolute hand, and contrast loss in single-particle electron cryomicroscopy. *J Mol Biol* 2003; **333**:721-745.

8 Chen S, McMullan G, Faruqi AR *et al.* High-resolution noise substitution to measure overfitting and validate resolution in 3D structure determination by single particle electron cryomicroscopy. *Ultramicroscopy* 2013; **135**:24-35.

9 Kucukelbir A, Sigworth FJ, Tagare HD. Quantifying the local resolution of cryo-EM density maps. *Nat Methods* 2014; **11**:63-65.

10 Zhou N, Wang H, Wang J. EMBuilder: A Template Matching-based Automatic Model-building Program for High-resolution Cryo-Electron Microscopy Maps. *Sci Rep* 2017; **7**:2664.

11 Emsley P, Lohkamp B, Scott WG, Cowtan K. Features and development of Coot. *Acta Crystallogr D Biol Crystallogr* 2010; **66**:486-501.

12 Afonine PV, Poon BK, Read RJ *et al.* Real-space refinement in PHENIX for cryo-EM and crystallography. *Acta crystallographica Section D, Structural biology* 2018; **74**:531-544.

13 DeLano WL. The PyMOL Molecular Graphics System. *on World Wide Web* [*http://wwwpymolorg*](http://wwwpymolorg) 2002.

14 Thompson JD, Gibson T, Higgins DG. Multiple sequence alignment using ClustalW and ClustalX. *Current protocols in bioinformatics* 2002:2.3. 1-2.3. 22.

15 Liu X, Zeng J, Huang K, Wang J. Structure of the mannose transporter of the bacterial phosphotransferase system. *Cell Res* 2019; **29**:680-682.

16 Frana TS. Investigation of microcin 24 for applications in food safety, mechanism of resistance and effect on activity after site-directed mutagenesis. *PhD thesis* 2004; **Iowa State University**.
